# Noise induced quiescence of epileptic spike generation in patients with epilepsy

**DOI:** 10.1007/s10827-020-00772-3

**Published:** 2021-01-08

**Authors:** Charith N. Cooray, Ana Carvalho, Gerald K. Cooray

**Affiliations:** 1 Department of Clinical Neuroscience, Karolinska Institutet, Stockholm, Sweden; 2 Clinical Neurophysiology, Karolinska University Hospital, Stockholm, Sweden; 3 Clinical Neurophysiology, Great Ormond Street Hospital for Children, London, UK

**Keywords:** Interspike interval, Cortical-state transitions, Spike threshold, EEG, Epilepsy

## Abstract

Clinical scalp electroencephalographic recordings from patients with epilepsy are distinguished by the presence of epileptic discharges *i.e.* spikes or sharp waves. These often occur randomly on a background of fluctuating potentials. The spike rate varies between different brain states (sleep and awake) and patients. Epileptogenic tissue and regions near these often show increased spike rates in comparison to other cortical regions. Several studies have shown a relation between spike rate and background activity although the underlying reason for this is still poorly understood. Both these processes, spike occurrence and background activity show evidence of being at least partly stochastic processes. In this study we show that epileptic discharges seen on scalp electroencephalographic recordings and background activity are driven at least partly by a common biological noise. Furthermore, our results indicate noise induced quiescence of spike generation which, in analogy with computational models of spiking, indicate spikes to be generated by transitions between semi-stable states of the brain, similar to the generation of epileptic seizure activity. The deepened physiological understanding of spike generation in epilepsy that this study provides could be useful in the electrophysiological assessment of different therapies for epilepsy including the effect of different drugs or electrical stimulation.

## Introduction

Epilepsy is a chronic disorder characterized by heterogeneous and dynamic pathophysiological processes leading to an altered balance between excitatory and inhibitory influences at cortical level. The disease involves a network of regions of the brain which is specific to each patient.

Electroencephalography (EEG) of patients with epilepsy is characterized by the presence of epileptic discharges, *e.g.* spikes or sharp waves. Hereafter, we will use the word spike to designate all epileptic discharges on the EEG. In clinical recordings spikes often repeat randomly on a background of fluctuating potentials (*i.e.* the spontaneous EEG activity) although their frequency might be affected by level of alertness or wakefulness, as well as the epileptogenicity of the underlying tissue. Spike rate has in several studies been shown to reduce during electrical stimulation of the cortex together with an attenuation of the spontaneous EEG activity (Kinoshita et al. 2005, Westin et al. 2019, Yamamoto et al. 2006). The underlying cause of this change and the relation between background attenuation and spike rate is still not understood. The spike waveform has been modelled using several computational models both for spikes seen in single neuron measurements and clinical spikes seen in epilepsy. Single neuron spiking has been effectively modelled using the Hodgkin-Huxley model which models both sodium and potassium ion channelling (Hodgkin and Huxley 1952). Macroelectrode recordings, as used in clinical settings (EEG), measure potentials generated by postsynaptic potentials in the dendritic trees of cortical pyramidal cells. Synchronous activity from 10,000–100,000 cells are required to induce measurable potentials. Cortical histology and functional structure indicate presence of cortical columns arranged in microcircuits containing pyramidal cells, excitatory and inhibitory interneurons (Douglas et al. 1989). A patch of cortex generating an EEG signal can be approximated as a collection of cortical columns. Several computational models of cortical microcircuits/columns exist which have been shown to generate both spontaneous EEG activity and spikes (David and Friston 2003; Jansen and Rit 1995; Pinotsis et al. 2012; Wendling et al. 2000; Wilson and Cowan 1972). Single cell and population induced spikes can often be approximated using phenomenological models, where the FitzHugh-Nagumo model is one of the simplest (FitzHugh 1961; Nagumo et al. 1962). The mentioned models are all deterministic and cannot model randomly occurring spikes if not modified. Adding random noise to the mentioned models; however, allows for randomly generated spikes. Background activity of EEG, similar to spike rate, is dependent on the state of the brain and characterized by different rhythms with a unique spectral distribution (Schomer and Da Silva 2012). However, the exact prediction of EEG potentials over time is extremely difficult due to the high level of randomness in the signal. Perturbation of the mentioned models with noise generate signals with frequency characteristics similar to measured background EEG data.

In this study we hypothesize that epileptic discharges seen on scalp EEG and background activity are driven at least partly by a common biological noise. Our objective will be to investigate the relation between interspike interval (ISI) duration, background activity and a latent noise variable. We will show data that indicates that the interspike interval distribution is dependent on the noise intensity together with a maxima of ISI for intermediate noise levels.

## Method

### Patients

Data was collected from 19 patients undergoing prolonged EEG recordings at the epilepsy monitoring unit at the Department of Paediatric Neurology at Karolinska University Hospital, Stockholm, Sweden. Median age was 7 (1–18) years and 11 were male.

### EEG recordings

Patients were all recorded with 21 scalp-electrodes according to the 10–20 system (Fp1, Fp2, F7, F3, Fz, F4, F8, T3, C3, Cz, C4, T4, T5, P3, Pz, P4, T6, O1, O2, A1 and A2). Recordings were performed using a NicoletOne EEG System (Natus, Pleasanton, CA 94566 USA). Data was registered at 250 Hz and bandpass filtered between 0.5 – 70 Hz. Each patients was recorded during 1–7 days. All EEG data was analysed by an Electroencephalographer (AC) identifying ictal (seizure) and interictal (non-seizure) data. All recordings with ictal episodes were removed from further analysis. Interictal EEG data was further divided into awake and sleep states, with separate analysis for each state of consciousness. Average amount of data per patient for each state was 8.0 h of wakefulness and 11.9 h of sleep. Data was divided into wake and sleep states using visual analysis of the EEG with identification of state specific waveforms and spectral distributions unique to each state (Schomer and Da Silva 2012). Visual analysis of 1–2 h of awake and sleep data was performed to identify epileptic discharges. The data was dominated by either focal or regional spikes for each patient. The interictal discharges of each patient was located using a spike-detection algorithm (Barkmeier et al. 2012). The threshold of the algorithm was set to an optimal level (with maximal Youden’s J statistic) for each patient to improve detection of epileptic discharges as has been described previously (Westin et al. 2019).

### Data analysis

All analysis was done separately for awake and sleep data with an average reference (*i.e.* 0 mean over all electrodes). Please see Appendix for exact definition of parameters (including ${\mathcal{G}}_x^i,{\mathcal{J}}^i$). Parameters were normalized as necessary to allow for merger of data values across patients in the subsequent analysis.

#### ISI-analysis

**Fig. 1:**
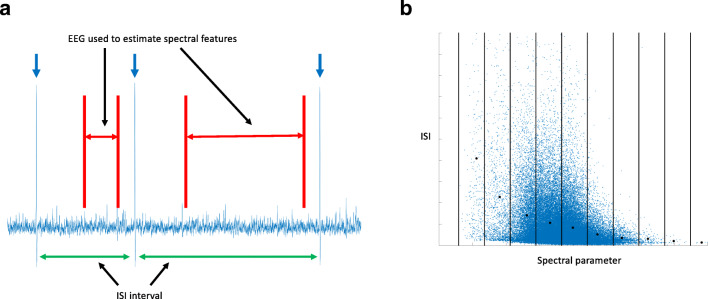
A. Schematic of data processing. Spikes were identified and marked at the peak potential using an algorithm (blue arrows). Time between spikes was measured to define the interspike interval (green arrows). EEG data between the spikes was used to estimate spectral parameters for the interspike interval, marked in red. EEG data 1 s after and 100 ms before a spike maximum was removed from the spectral analysis. This was done to prevent spike waveforms contaminating the analyzed EEG data. B. Schematic of spectral parameter averaging. The blue dots are individual parameter values from interspike intervals (${\mathcal{G}}_x^i,{\mathcal{J}}^i$). To further delineate any underlying relation between spectral and ISI parameters the data was subdivided along the abscissa (black lines) into segments. The data (blue dots) in each segment was averaged for both parameters (spectral and ISI) and plotted as a black dot. Further analysis was done on the new set of reduced data ($\overline{{\mathcal{G}}_x^i}$and $\overline{{\mathcal{J}}^i}$, *i.e.* the black dots)

Data was divided into windows (interspike interval) limited by interictal epileptic discharges, see Fig. 1. The ISI (*I*^*i*^) was defined as the length of the window in seconds. The ISI was normalized for each patient (${\mathcal{J}}^i$) for awake and for sleep data such that the mean was 1 for both awake and sleep data for each patient.

#### Spectral analysis

EEG data from each interspike interval was used to estimate the window specific background activity. EEG data 1 s after the maximum of the first spike and 100 ms before the limiting spike was removed from the window to remove direct effects of spike waveforms on subsequent EEG activity, see Fig. 1. Each segment of EEG data was further subdivided into 1 s epochs to perform spectral analysis of the signal. All analysis was done in the following frequency bands (1 ≤ *f* < 40, 1 ≤ *f* < 4, 4 ≤ *f* < 8, 8 ≤ *f* < 12, 12 ≤ *f* < 40 Hz). Cross spectral density was estimated for each interspike interval window for the different frequency bands and normalized (${\mathcal{G}}_x^i$). Analysis included visualization of scatter plots between ISI and spectral parameters. To further reveal any relation between the variables not clearly visible on the scatter plots the range of the “independent” variable (*e.g.* ${\mathcal{G}}_x^i$) was divided into 100 sub-intervals. Within each sub-interval the average of the dependent and independent variable was calculated ($\overline{{\mathcal{G}}_x^i}$ and $\overline{{\mathcal{J}}^i}$). The resulting data was used for visual analysis and log-log plotting to reveal quantitative relations between the variables. This analysis is schematically illustrated in Fig. 1.

### Computational modelling

#### Background activity

The background activity was approximated as the stationary state of the perturbation of a neural mass model (*H,* n coupled ordinary differential equations) under noise (see Moran et al. 2009 for details on a similar description). A stable point was chosen as the point of perturbation. The noise term was given by a standard n-dimensional Brownian motion with covariation matrix ***σσ***^*T*^ and zero mean, *d****B***_*t*_. The dynamics was governed by the following stochastic differential equation:

$$ d\boldsymbol{x}=H\left(\boldsymbol{x}\right) dt+\boldsymbol{\sigma} d{\boldsymbol{B}}_t $$

$$ \boldsymbol{x}\in {\mathrm{\mathbb{R}}}^n,\boldsymbol{\sigma} \in {\mathrm{\mathbb{R}}}^n\times {\mathrm{\mathbb{R}}}^n $$

The above function *H* was linearized around a stable point (as we are studying perturbations) giving,

$$ d\boldsymbol{x}={L}_1\left(\boldsymbol{x}\right) dt+\boldsymbol{\sigma} d{B}_t $$

The spectral density of ***x***, can then be shown to be proportional to the covariance matrix of the noise (Wiener-Khintchine Theorem),

$$ S\left({\boldsymbol{\omega}}_{\boldsymbol{x}}\right)\propto {\boldsymbol{\sigma} \boldsymbol{\sigma}}^T $$

The measured EEG signals over the m electrodes, ***y***(*t*) ∈ *ℝ*^*m*^, was approximated by a linear mapping of the state variables (***x***) under some function ***L***_**2**_ ∈*ℝ*^*n*^ × *ℝ*^*m*^.

$$ \boldsymbol{y}(t)={\boldsymbol{L}}_{\mathbf{2}}\left(\boldsymbol{x}\right) $$

The spectral density of y would then be given by the following,

$$ S\left({\boldsymbol{\omega}}_{\boldsymbol{y}}\right)\propto {{\boldsymbol{L}}_{\mathbf{2}}}^T{\boldsymbol{\sigma} \boldsymbol{\sigma}}^T{\boldsymbol{L}}_{\mathbf{2}} $$

Due to the above relation the spectral density of the EEG will be used as a proxy for the input noise to the cortex.

#### Spike generation

The FitzHugh-Nagumo model with *I = 0.326* and noise was used to study the generation of spikes (Tuckwell et al. 2003). *X*_*t*_ is the voltage variable and *Y*_*t*_ the recovery variable. The noise term is given by standard Brownian motion with correlation matrix *σ*^2^ and zero mean, *dB*_*t*_.

$$ d{X}_t=\left({X}_t-\frac{{X_t}^3}{3}-{Y}_t+\kern0.5em I\ \right) dt+\sigma d{B}_t $$

$$ d{Y}_t=0.08\left({X}_t-0.8{Y}_t+0.7\right) dt+\sigma d{B}_t $$

**Fig. 2:**
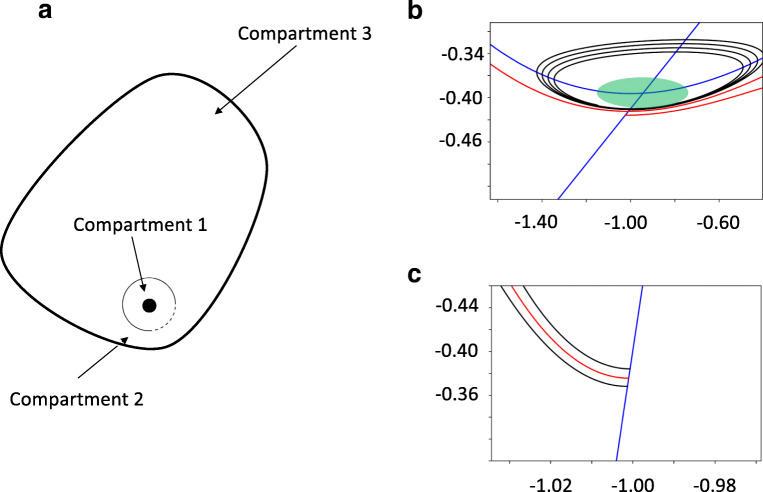
A. Schematic figure of phase plane of FitzHugh-Nagumo model together with the three compartments used for a model amenable for computational estimation of ISI. The dotted regions indicate regions where the trajectories transferred between compartments. B. Compartment 1 shaded in green. The two blue lines are the nullclines of the FitzHugh-Nagumo model with the stable fixed point located at the intersection. The black spiral trajectory shows a particle moving towards the stable fixed point. The red line indicates trajectory moving along the limit cycle. C. Compartment 2 was located along the limit cycle near the stationary point where trajectories moved to the latter

**Fig. 3:**
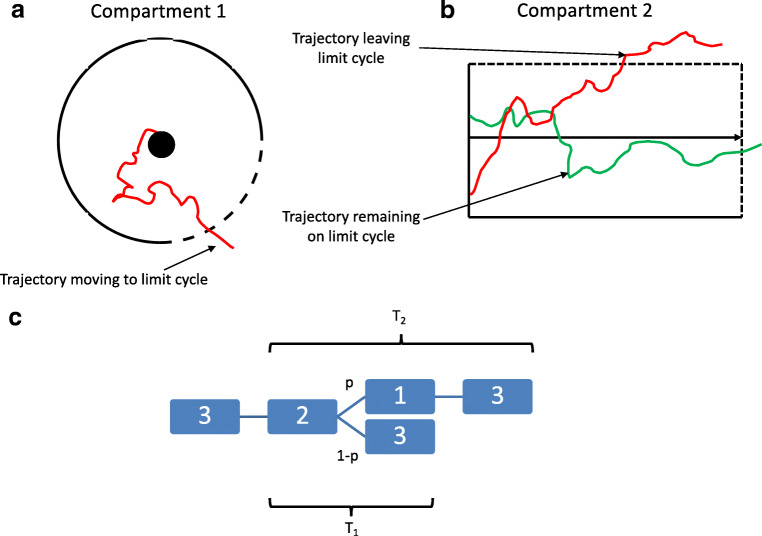
A. Compartment 1 after rescaling with the stationary point at the center. An absorbing boundary was placed in the lower right quadrant and a reflecting over the other quadrants. The red line shows a trajectory leaving compartment 1 and then transferring to compartment 3. B. Compartment 2 after rescaling. An absorbing boundary was placed at the top and right boundary. Reflecting boundaries were placed over the bottom and left boundary. The red trajectory is absorbed by the top boundary and then transferred to compartment 1. The green trajectory is absorbed by the right boundary and transferred to compartment 3. C. Schematic figure of the compartments and the transitions between them and the probabilities of these. Compartment chain 2, 1 and 3 is represented by 1 spike as is compartment chain 2 to 3. The time for these compartment chains T_1_ and T_2_ was estimated in the appendix. The expected ISI was estimated using these two times and p, the probability for transition from compartment 2 to 1

In this setting the dynamics of the FitzHugh-Nagumo is described by a stable stationary state surrounded by a stable orbit. The trajectories moving along the stable orbit represented spiking of the model where one revolution resulted in a spike-like waveform of *X*_*t*_ when plotted against time (*t*), see Fig. 2. To analyse inter-spike intervals the movement of trajectories between the stable point and the stable limit cycle was analysed. To make the estimation amenable for computational analysis further simplification was done. The phase space was compartmentalised into three regions, Fig. 2. The first described trajectories moving around the stable point and this motion was modelled by movement within a disc surrounding the stable point. The lower right quadrant of the circle limiting the disc was modelled as an absorbing boundary and the remaining boundaries as reflecting, Fig. 3. Trajectories that reached the absorbing boundary represented transitions to the stable orbit of the compartmentalised model. Motion along the limit cycle was modelled using two compartments, 2 and 3. The trajectories were allowed to make a transition to the first compartment (from the limit cycle) in the second compartment but not in the third compartment. The third compartment represented flow on the limit cycle away from the stable point (where the probability of transition between the compartments was low). The second compartment represented an area where the transition to the stable point was of higher probability due to the proximity between the stable limit cycle and fixed point. The mean escape time was estimated from all three compartments. The timing between two inter-spikes would then consist of the following sequence of events. Trajectories in compartment 2 could either move to compartment 1 or 3. We estimated the transition probabilities between the compartments and the mean time spent in each as described in the appendix, Fig. 3.

We estimated the expected ISI, *E*[*T*], for all possible transition chains. *T* denoted ISI for a trajectory. *μ* denotes the measure of trajectories in phase space. *φ* denotes a trajectory. *p* denotes the probability of transfer from compartment 2 to 1. *T*_1_ denotes the expected time spent in the following chain of transitions: compartment 3–2-3. *T*_2_ denotes the expected time spent in the following chain of transitions: compartment 2–1-3.

$$ E\left[T\right]=\int d\mu \left(\varphi \right)T\left(\varphi \right) $$

$$ E\left[T\right]=\underset{n\to \infty }{\lim}\sum \limits_{k=0}^n\frac{\left[\left(n-k\right){T}_1+k{T}_2\right]}{n}\frac{n!}{k!\left(n-k\right)!}{\left(1-p\right)}^{n-k}{p}^k $$

$$ E\left[T\right]\approx \underset{n\to \infty }{\lim}\int dk\left[{T}_1+\frac{k}{n}\left({T}_2-{T}_1\right)\right]\ \mathcal{N}\left( np, np\left(1-p\right)\right) $$

$$ E\left[T\right]\approx \underset{n\to \infty }{\lim}\int dx\left[{T}_1+x\left({T}_2-{T}_1\right)\right]\mathcal{N}\left(p,{\sigma}_n\right) $$

$$ E\left[T\right]\approx \int dx\left[{T}_1+x\left({T}_2-{T}_1\right)\right]\delta \left(x-p\right)=\left(1-p\right){T}_1+p{T}_2 $$

On the third line we approximated a binomial distribution with a Gaussian distribution. We used the Fokker Planck equation associated with the FitzHugh-Nagumo model in each compartment to estimate the following parameters for different noise levels (see appendix 7.3): *p*, *T*_*1*_ and *T*_*2*_. The partial differential equations were solved using FENICS (Alnaes et al. 2015).

#### Spectral parameters and input noise

As shown in the *Background activity* noise intensity is proportional to spectral density. In the following analysis we will use spectral density parameters as proxies for noise intensity.

## Results

### ISI-distribution

**Fig. 4:**
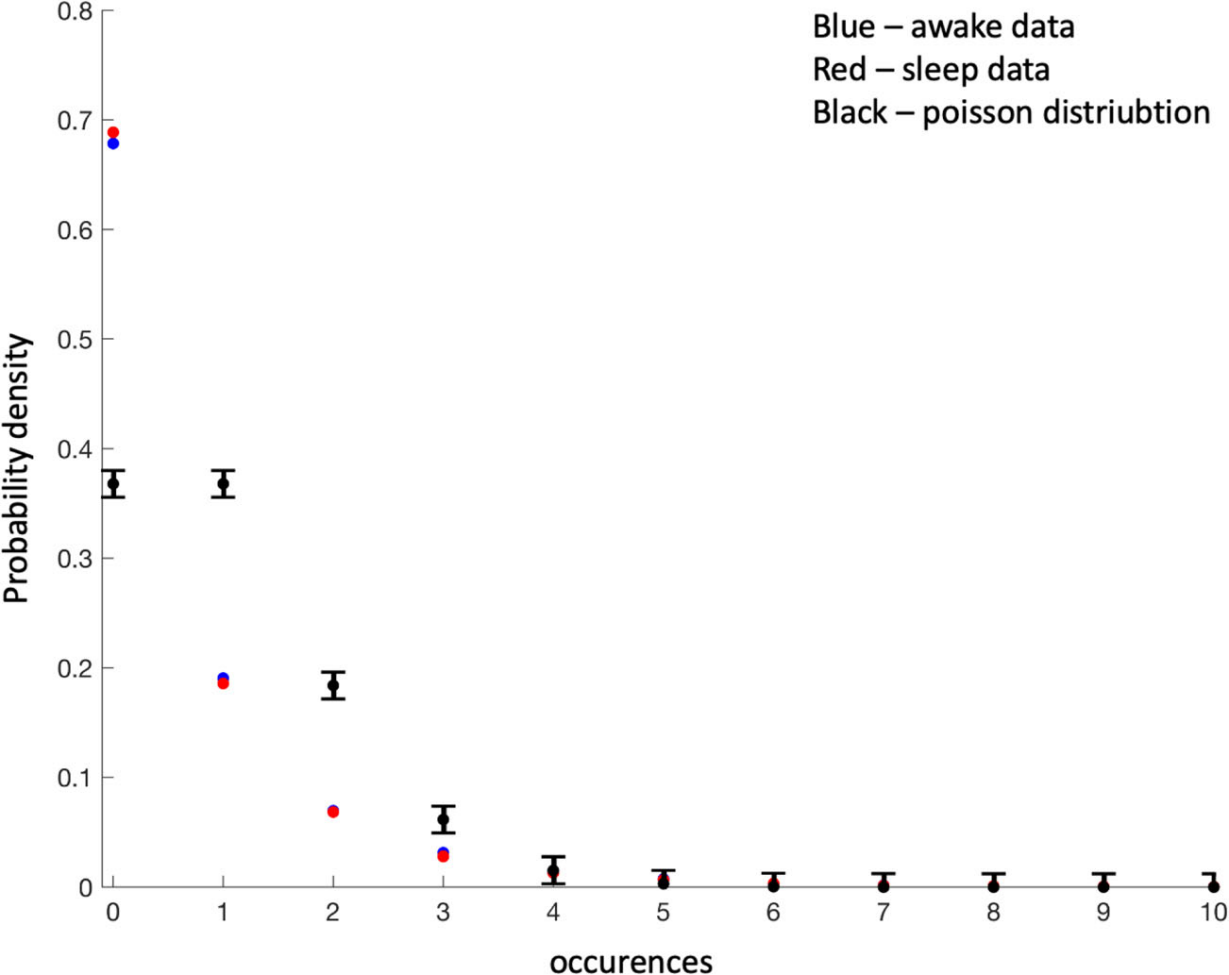
Poisson distribution with rate factor 1 in black with 3 standard deviation interval marked as a black line. Red and blue show the corresponding distribution of the spike rate for awake and sleep data. Note that the data was normalized among patients where mean spike rate for each patient was set to 1

ISI was measured (*I*^*i*^) and normalised (${\mathcal{J}}^i$) for each patient as described in *Method* and *Appendix.* The probability distribution for awake and sleep data were similar and no significant changes could be found. The distribution of normalised ISI (${\mathcal{J}}^i$) was used to estimate the number of spikes occurring in one unit of time both for awake and sleep data. Note that the data was normalized among patients where mean spike rate for each patient was set to 1. The resulting distribution was compared to the Poisson distribution with rate 1, Fig. 4. The measured spike occurrences (awake and sleep data) were more than three standard deviations from the Poisson distribution, indicating a significant difference.

### Cross spectral density

Averaged cross spectral density (${G}_x^i$) was estimated for each frequency band as described in *Method* and *Appendix.* This was done separately for awake and sleep data and no significant differences between awake and sleep were found.

#### Spectral parameters and interspike interval

**Fig. 5:**
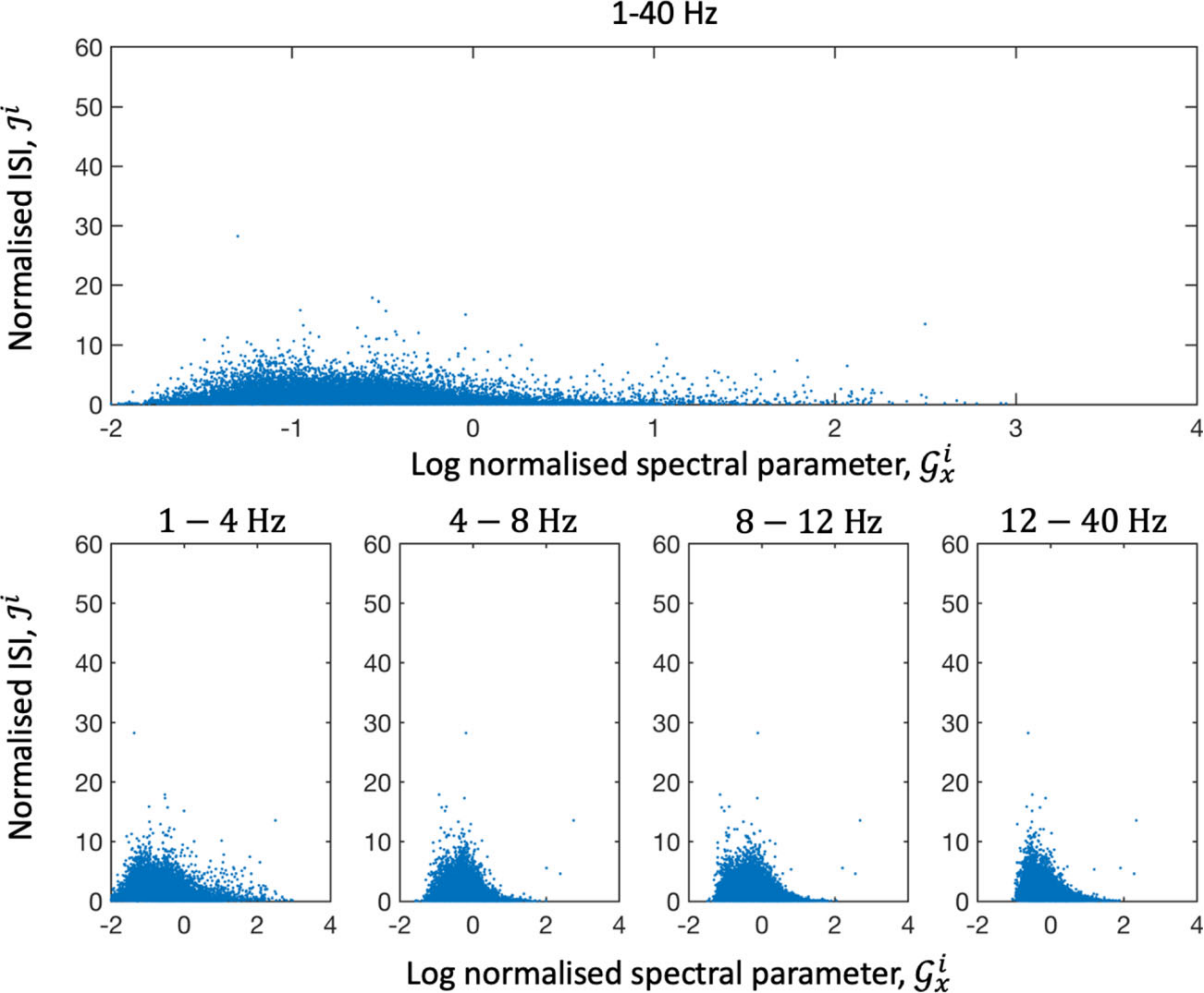
Scatter plots between the logarithm of the normalized cross spectral density (${\mathcal{G}}_x^i$) and ISI (${\mathcal{J}}^i$) for awake data

The spectral parameters were assessed using normalized cross spectral density (${\mathcal{G}}_x^i$). The parameters were estimated for each interspike interval window which allowed us to create scatter plots between the spectral parameters and normalized ISI (${\mathcal{J}}^i$). This was done separately for awake and sleep data. Scatter plots of normalized cross spectral density (${\mathcal{G}}_x^i$) and ISI showed Gaussian distributions with a right sided tail with increased ISI around −1 for the full frequency band (1-40 Hz) and around −1 to 0 for the other frequency bands (1–4, 4–8, 8–12 and 12–40 Hz) for both awake (Fig. 5) and sleep data.

**Fig. 6:**
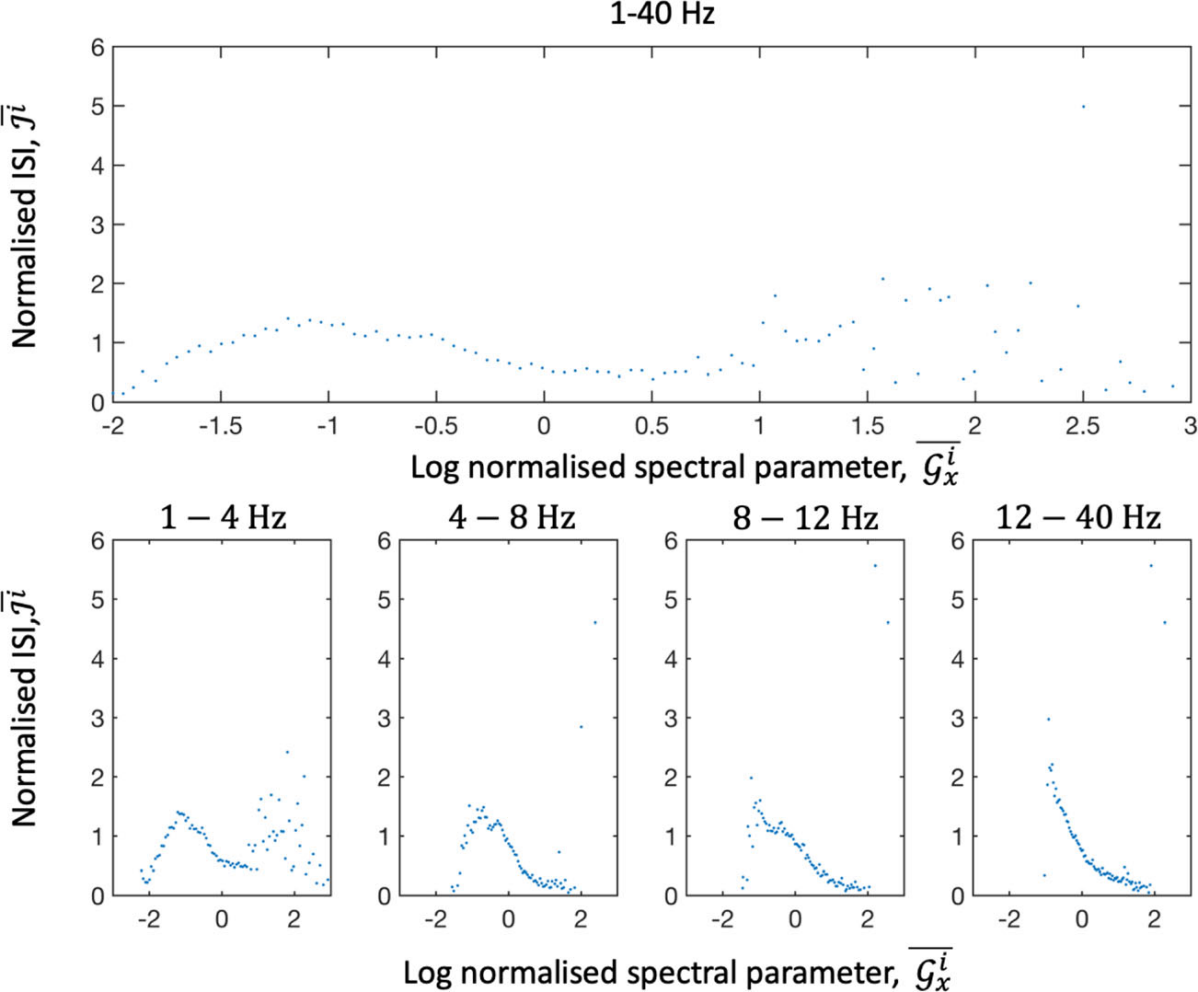
Plots between the logarithm of the mean normalized cross spectral density ($\overline{{\mathcal{G}}_x^i}$) and mean ISI ($\overline{{\mathcal{J}}^i}$) for awake data. In the 4–8, 8–12 and 12–40 Hz band there was a decreasing curve as the spectral parameter increased. At low values of the spectral parameter there was evidence of a maxima (all bands except 12-40 Hz)

The mean values of normalized cross spectral density and interspike interval ($\overline{{\mathcal{G}}_x^i},$ and $\overline{{\mathcal{J}}^i}$) was calculated as described in *Method.* Awake activity within 1–4 Hz, 4–8 Hz and 8–12 Hz and the full bandwidth showed maximal values for ISI for intermediate noise levels. The 12-40 Hz frequency band indicated a monotonically decreasing curve (Fig. 6). Sleep data showed similar results to awake data.

Log normalized cross spectral density was plotted against log ISI. There was an approximate linear relationship between both variables between −0.5 to 1 (awake) and − 1 to 1 (sleep). This was evident for all frequency bands but most clear for 4–8, 8–12 and 12–40 Hz. The gradient was estimated for each frequency band for both awake and sleep data and varied between [−0.56,-0.19], Table 1.

**Table 1.** Gradient and intersect of linear regression between logarithm of spectral parameter (normalized cross spectral density) and logarithm of normalized inter-spike interval duration (ISI)

Frequency (Hz)	1--40	1--4	4--8	8--12	12--40
sleep					
gradient intersect	-0,2010 -0,0744	-0,2013 -0,0782	-0,3813 -0,1505	-0,3475 -0,1446	-0,4158 -0,1275
awake					
gradient intersect	-0,2050 -0,2346	-0,1933 -0,2263	-0,7189 -0,0700	-0,4163 -0,0544	-0,5583 -0,1318

### Computed ISI for FitzHugh-Nagumo model

**Fig. 7:**
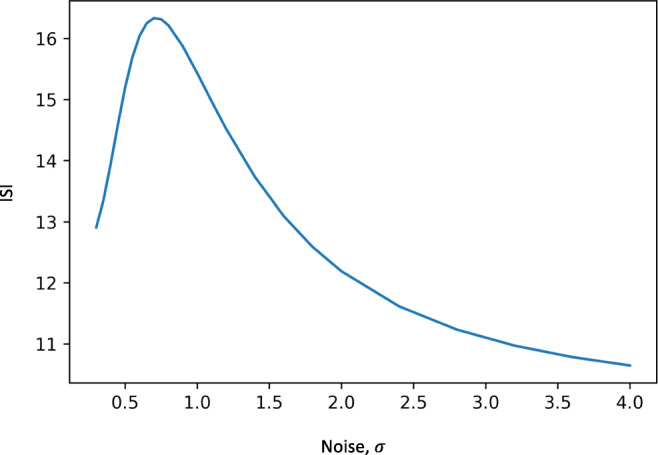
The graph of ISI estimated from the stochastic Fitzhugh-Nagumo model after compartmentalizing for different noise intensities. There is a quiescence of spike rate for intermediate noise intensities. Similar, curve was achieved after simulating the FitzHugh-Nagumo model

As described in methods the ISI was computed for different noise intensities for the FitzHugh-Nagumo model. There was a maximal ISI interval for intermediate noise intensities with reducing ISI for small and large noise levels, Fig. 7.

## Discussion

This study investigated the relation between epileptic interspike intervals and a latent noise variable in patients with epilepsy. The noise intensity was shown to be proportional to the spectral density of the EEG recording, see *Computations*. We found that interspike interval or duration was dependent on the latent noise variable and that ISI was maximal for intermediate noise levels. This behavior has not previously, to our knowledge, been presented or noted in clinical EEG. The FitzHugh-Nagumo model showed similar results for ISI in relation to noise level and we will discuss why our results could indicate that epileptic spikes are created by the transition between different states of the cortex (or brain) similar to epileptic seizures.

The normalized interspike intervals measured from the patients were used to plot a histogram over number of occurrences of spikes per unit time. If the average spike rate was constant this distribution would follow a poisson distribution which we could show it did not. Data from both awake and sleep states showed a clear statistical difference from the poisson distribution. This could indicate a process where the spike rate was dependent on an underlying non-constant parameter resulting in a varying average spike rate or equivalently varying interspike intervals. Computational modelling of spike triggering requires the addition of external (biological) noise and it has been shown that average spike rate changes with noise intensity. The system is randomly driven to a fixed threshold by the noise such that increasing noise intensity reduces ISI (Tuckwell et al. 2009; Tuckwell et al. 2003; Tuckwell and Wan 2005). We computed the ISI for the FitzHugh-Nagumo model (with noise) showing, as has been previously presented in the literature, that average spike intervals are a function of the noise intensity driving the system. To find evidence of a similar relation in our clinical data, we would need to estimate the biological noise driving the system. The analyzed data is from an *in-vivo* system (patient data) with no ethical possibilities of controlling the incoming noise or directly measuring it. Instead we relied on the relation between the spectral distribution of the data and the driving noise. In 2.4.1, we suggested that the spectral density of the EEG data could be approximated as being proportional to an undetermined biological noise process driving the system similar to what has been modeled in several publications (Moran et al. 2009; Robinson et al. 2001; Steyn-Ross et al. 1999). Based on these previous studies and our own computation we estimated that there could be a proportionality between the spectral parameters estimated in the study and the latent biological noise intensity. This allowed us to have an estimate of noise intensity in the form of the spectral parameters that we estimated for the data. As was shown in Results, the ISI-spectral parameter curves showed a maxima at a log normalized spectral parameter of around −1 for the different frequency bands indicating that the findings were at least partly frequency independent. The maxima is not typical for classical stochastic resonance which is frequency dependent (McDonnell et al. 2015). Assuming that the spectral features were the result of fluctuations around a stable point the independence on the frequency band would be expected as discussed in Section 2.4.1. and could also be estimated from the ISI simulation in the FitzHugh-Nagumo model. The spectral “EEG” parameters of the FitzHugh-Nagumo model would then be estimated from the fluctuations of the trajectories around the stable steady point. The assumption that spontaneous EEG activity is generated by the fluctuation around a stable point is a simplification of a complex neurobiological process which does not allow the activities within the different frequency bands of spontaneous EEG to be modelled as independent processes. In the present study this simplifying assumption was supported by the results as it was not possible to distinguish differences in the relation between ISI and spectral parameters for different frequency bands. If future studies reveal frequency band dependency on the ISI maxima more complex models would be required. The noise induced ISI maximum has been seen for single and multi-neuron measurements both *in vitro* and *in vivo* (Buchin et al. 2016; Gluckman et al. 1998). Simulations and computations have shown similar findings for a multitude of spike models including both the Hodgkin-Huxley and FitzHugh-Nagumo model (Tuckwell et al. 2009; Tuckwell et al. 2003; Tuckwell and Wan 2005).

The phase space of the Fitz-Hugh-Nagumo model was simplified to allow for computation of the ISI. We used three compartments to describe the flow of trajectories: compartment 1 contained the stable fixed point and compartment 2 and 3 contained the stable limit cycle. At low noise levels the trajectories were shown to collect along the stable limit cycle. As the noise level increased the probability of getting attracted to the stable fixed point increased which resulted in an increase in ISI as trajectories were “held” at the stationary point. With further increasing noise, the time where trajectories were located around the fixed point (compartment 1) reduced resulting in a reduction of ISI. Removal of the limit cycle from the FitzHugh-Nagumo model (by changing the parameter *I*) resulted in ISI values decreasing with increasing noise (simulated data). We further studied the effect of varying the geometry of the compartments which did not change the qualitative relation between ISI and noise. The results seen in the FitzHugh-Nagumo model resemble the findings we see in epileptic spikes in patients with epilepsy. The analogy with the FitzHigh-Nagumo model (and several other models in the literature) would indicate that epileptic spikes are created by the transition between two stable or semi-stable states of the brain.

## Conclusion

This study has shown the effect of a latent noise variable on epileptic spike production in patients with epilepsy indicating the possible transition of the cortex between semi-stable states, similar to the generation of epileptic seizure activity (Jirsa et al. 2014). We hypothesize that these findings are general and should be reproduced in other studies of epileptic spikes in patients with epilepsy including different modalities such as MEG and intracranial EEG. Further study, including spikes and seizure activity and their dynamics could be of importance in defining the brain states involved in epileptic activity (*i.e.* epileptic spikes and seizure activity). The deepened understanding of the electrophysiology of epilepsy that this study provides could be useful in the assessment of different therapies for epilepsy including the effect of different drugs or electrical stimulation.

## Appendix

### Definition of normalized ISI (${\mathcal{J}}^{\mathrm{i}}\Big)$

The ISI (*I*^*i*^) was defined as the length of the window in seconds. The normalized interval (${\mathcal{J}}^i$) was defined as follows, where N denotes the number of interspike interval windows:

$$ {\mathcal{J}}^i=\frac{I^i}{\frac{1}{N}{\sum}_{j=1}^{j=N}{I}^j} $$

### Definition of normalized cross spectral density (${\mathcal{G}}_{\mathrm{x}}^{\mathrm{i}}$)

The cross spectral density, ${G}_{xy}^{ij}\left(\omega \right)$, where *x* and *y* denote EEG channels, was calculated for each interspike interval window (i) and epoch (j) (each interspike interval window was subdivided into 1 s long epochs).

$$ {G}_{xy}^{ij}\left(\omega \right)=\left|{F}_x^{ij}\left(\omega \right){F}_y^{ij}{\left(\omega \right)}^{\ast}\right| $$

Where ${F}_x^{ij}\left(\omega \right)$ is the fourier transform of EEG channel x in the j-th epoch of the i-th interspike interval window. The average cross-spectral density was estimated as below for each interspike interval (i) and for the channel (x) with maximum spike amplitude; n denotes number of EEG-channels and m denotes number of epochs in the i-th interspike interval window:

$$ {G}_x^i\left(\omega \right)=\frac{1}{n}\sum \limits_y\frac{1}{m}\sum \limits_j{G}_{xy}^{ij}\left(\omega \right) $$

The averaged cross spectral density was estimated in the following frequency bands: 1–40, 1–4, 4–8, 8–12 and 12–40 Hz. This was done as follows:

$$ {\int}_1^{40}{d\omega G}_x^i\left(\omega \right)={G}_x^i\left(1,40\right) $$

The averaged cross spectral density, ${G}_x^i$, was normalized (${\mathcal{G}}_x^i$) for each patient to allow for merger of data values across patients in the subsequent analysis:

$$ {\mathcal{G}}_x^i=\frac{G_x^i}{\frac{1}{N}{\sum}_{j=1}^{j=N}{G}_x^j\left(1,40\right)} $$

This was estimated for the different frequency bands: 1–40, 1–4, 4–8, 8–12 and 12–40 Hz:

$$ {\int}_1^{40}{d\omega \mathcal{G}}_x^i\left(\omega \right)={\mathcal{G}}_x^i\left(1,40\right) $$

### Estimation of the relation between inter-spike interval and input noise in the FitzHugh-Nagumo model

The Fokker Planck operator was used to estimate the expected time of trajectories in each compartment and the following transition probabilities: compartment 2 to 1 and compartment 2 to 3.

### Estimation of the expected time spent in compartment 1

An ellipse was used to model compartment 1 which was centered at the stationary state of the system (−0.9719, −0.3399), Fig. 2.

$$ {\left(\frac{\overline{x}+0.9719}{0.12}\right)}^2+{\left(\frac{\overline{y}+0.3399}{0.03}\right)}^2<1 $$

An absorbing boundary was placed on the lower right quadrant of the boundary and a reflecting on the rest of the boundary. Rescaled and translated dimensions were used $\left(x=\frac{\overline{x}+0.9719}{0.12},y=\frac{\overline{y}+0.3399}{0.03}\right)$ such that the ellipse was transformed into a disc with centre at the origin, Fig. 3. The dynamics of the trajectories within compartment 1 was given by the following stochastic differential equation:

$$ d{X}_t=\left(-0.0048{X_t}^3+0.0389{X_t}^2+0.6851{X}_t-0.25{Y}_t+0.00013\right) dt+8.3333\sigma d{B}_t $$

$$ d{Y}_t=\left(0.32{X}_t-0.064{Y}_t+0.00005\right) dt+33.3\sigma d{B}_t $$

The expected escape time *f(x)* starting at *x* from compartment 1 was estimated using Dynkin’s formula (Ch7. Oksendal 2013). Where *f(x)* satisfies the following partial differential equation (PDE),

$$ {\displaystyle \begin{array}{c}L=\left[-0.0048{x}^3+0.0389{x}^2+0.6851x-0.25y+0.00013\right]\frac{\partial }{\partial x}+\left(0.32x-0.064y+0.00005\right)\frac{\partial }{\partial y}+\frac{\sigma^2}{2}\left(69\frac{\partial^2}{{\partial x}^2}+1109\frac{\partial^2}{{\partial y}^2}\right)\\ {}-1= Lf(x)\\ {}\begin{array}{c}{\left.0=f(x)\right|}_{{\mathrm{\partial \Omega}}_a}\\ {}{\left.0={\partial}_nf(x)\right|}_{{\mathrm{\partial \Omega}}_r}\end{array}\end{array}} $$

Note, that *L* is the adjoint of the Fokker Planck operator for the stochastic differential equation governing the trajectories. Boundary conditions included an absorbing boundary limiting the lower right quadrant (∂Ω_*a*_) and reflecting on the remaining boundary (∂Ω_*r*_). The PDE was solved using FENICS and the expected time in compartment 1 was estimated with *x* located at the stationary point (Alnaes et al. 2015).

#### Estimation of the expected time spent in compartment 2 and transition probabilities to compartments 1 and 3

Compartment 2 was located symmetrically around the stable limit cycle in the box *x* ∈ [−1.03, −1] and *y* ∈ [−0.35, −0.383]. The upper boundary was set at a distance of 0.015 from the stable limit cycle, which was the distance to the unstable limit cycle. A symmetrical lower boundary was placed at a the same distance from the stable limit cycle, 0.015, Fig. 2. The length of compartment 2 was set to 0.03. A coordinate system was set with one coordinate along the limit cycle (*X*) and one transverse (*Y*) to this. This compartment was rescaled into a unit-square, Fig. 3. The dynamics of the rescaled compartment 2 was given by,

$$ {\displaystyle \begin{array}{c}d{X}_t=2 dt+67\sigma d{B}_t\\ {}d{Y}_t=67\sigma d{B}_t\end{array}} $$

Note that we incorporated the factor of 70 in the noise term *σ* for the rest of this section. The Fokker Planck operator was given by (v = 2):

$$ {L}^{\dagger }=-v\frac{\partial }{\partial x}+\frac{\sigma^2}{2}\left(\frac{\partial^2}{{\partial x}^2}+\frac{\partial^2}{{\partial y}^2}\right) $$

The flow of the distribution of trajectories (p) was given by,

$$ J=-\left(\begin{array}{c}- vp+\frac{\sigma^2}{2}\frac{\partial p}{\partial x}\\ {}\frac{\sigma^2}{2}\frac{\partial p}{\partial y}\end{array}\right) $$

The following discretized equation was solved (with test function *q*),

$$ \int {qp}^{n+1}-q\Delta  t{L}^{\dagger }{p}^{n+1}-q{p}^n=0 $$

$$ I=\int {qp}^{n+1}-q\Delta  t\left(-v{\partial}_x{p}^{n+1}+\frac{\sigma^2}{2}\left(\frac{\partial^2}{{\partial x}^2}+\frac{\partial^2}{{\partial y}^2}\right){p}^{n+1}\right)-q{p}^n=0 $$

$$ {I}_1=\iint dx dy\ q\frac{\sigma^2}{2}\left(\frac{\partial^2}{{\partial x}^2}+\frac{\partial^2}{{\partial y}^2}\right){p}^{n+1}=\int dx\ \frac{\sigma^2}{2}q{\partial}_y{p}^{n+1}{\left|{}_{\mathrm{\partial \Omega}}^{y=1/2}-\int dx\ \frac{\sigma^2}{2}q{\partial}_y{p}^{n+1}\right|}_{\mathrm{\partial \Omega}}^{y=-1/2} $$

$$ +\int dy\ \frac{\sigma^2}{2}q{\partial}_x{p}^{n+1}{\left|{}_{\mathrm{\partial \Omega}}^{x=1/2}-\int dy\ \frac{\sigma^2}{2}q{\partial}_x{p}^{n+1}\right|}_{\mathrm{\partial \Omega}}^{x=-1/2} $$

$$ -\iint dxdy\ \frac{\sigma^2}{2}{\partial}_xq{\partial}_x{p}^{n+1}+\frac{\sigma^2}{2}{\partial}_yq{\partial}_y{p}^{n+1} $$

$$ =-\int dx\ \frac{\sigma^2}{2}q{\partial}_y{p}^{n+1}{\left|{}_{\mathrm{\partial \Omega}}^{y=-1/2}-\int dy\ \frac{\sigma^2}{2}q{\partial}_x{p}^{n+1}\right|}_{\mathrm{\partial \Omega}}^{x=-1/2} $$

$$ -\iint dxdy\ \frac{\sigma^2}{2}{\partial}_xq{\partial}_x{p}^{n+1}+\frac{\sigma^2}{2}{\partial}_yq{\partial}_y{p}^{n+1} $$

Robins conditions at reflecting boundaries (null probability flux),

$$ {\left.0=- vp+\frac{\sigma^2}{2}\frac{\partial p}{\partial x}\right|}_{x=-1/2} $$

$$ {\left.0=\frac{\sigma^2}{2}\frac{\partial p}{\partial y}\right|}_{y=-1/2} $$

$$ {\left.{I}_1=-\int dy\  qv{p}^{n+1}\right|}_{\mathrm{\partial \Omega}}^{x=-1/2} $$

$$ -\iint dxdy\ \frac{\sigma^2}{2}{\partial}_xq{\partial}_x{p}^{n+1}+\frac{\sigma^2}{2}{\partial}_yq{\partial}_y{p}^{n+1} $$

$$ I=\int {qp}^{n+1}+q\Delta  tv{\partial}_x{p}^{n+1}-q{p}^n\kern0.75em -{\Delta  tI}_1 $$

The final variational form was given by,

$$ {\left.I=\int {qp}^{n+1}+q\Delta  tv{\partial}_x{p}^{n+1}-q{p}^n\kern0.75em +\Delta  t\left(\frac{\sigma^2}{2}{\partial}_xq{\partial}_x{p}^{n+1}+\frac{\sigma^2}{2}{\partial}_yq{\partial}_y{p}^{n+1}\right)+\Delta  t\int dy\  qv{p}^{n+1}\right|}_{\mathrm{\partial \Omega}}^{x=-\frac{1}{2}}=0 $$

This variational form of the PDE was solved for p. The transition probability and expected time for transition to compartment 1 or 3 was estimated from p.

### Estimation of the expected time spent in compartment 3

In a similar way the expected time in compartment 3 was estimated.

### Estimation of expected time between spikes (ISI) for different levels of noise

*p*, *T*_*1*_ and *T*_*2*_ were estimated from the results of 7.2 and the ISI was calculated for different levels of noise, Fig. 7.
